# Contemporary Scientometric Characteristics of Obstructive Sleep Apnea

**DOI:** 10.7759/cureus.86079

**Published:** 2025-06-15

**Authors:** Mario Milkov, Dimitar Tomov, Miroslav Stoykov, Silvena Baycheva, Elena Todorova

**Affiliations:** 1 Faculty of Dental Medicine, Medical University, Varna, Varna, BGR; 2 Faculty of Medicine, Medical University, Varna, Varna, BGR

**Keywords:** databases, dynamic science internationalization, obstructive sleep apnea, science stratification, scientometrics

## Abstract

Objective

The purpose of the present investigation is to analyze some essential scientometric features of the dynamic institutionalization and internationalization of contemporary research on obstructive sleep apnea, a common and socially significant respiratory sleep disorder affecting adults and children worldwide.

Methods

A retrospective, problem-oriented, title-word-based search was performed in the Science Citation Index Expanded, MEDLINE, and BIOSIS Citation Index of Web of Science, as well as in Scopus, for the period 2019-2023. The annual dynamics of the quantitative characteristics of several essential scientometric indicators were comparatively assessed.

Results

A total of 9,692 publications were abstracted in the Science Citation Index Expanded, 8,103 in Scopus, 7,421 in MEDLINE, and 3,130 in BIOSIS. Authors from 118, 165, and 74 countries had publications abstracted in SCI Expanded, Scopus, and BIOSIS, respectively. Additionally, publications were available in a total of 23 languages (mainly in Scopus and MEDLINE). Researchers from the USA and China strongly dominated the field. The journals with the most papers on the topic, the most cited articles, and the most productive authors and scientific institutions were identified. The Hirsch index values were relatively high. Clinical neurology, respiratory system, and neurosciences were among the most common thematic categories in the Science Citation Index Expanded, while complications, polysomnography, diagnosis, therapy, and epidemiology of obstructive sleep apnea were the most prevalent Medical Subject Headings in MEDLINE.

Conclusion

The dynamic stratification of science in this field, in terms of publication output and international collaboration, deserves special attention from science policymakers and research managers in smaller countries.

## Introduction

Obstructive sleep apnea (OSA) is a common respiratory sleep disorder, associated with increased mortality, reduced quality of life, and a higher risk of work-related accidents and injuries [1].

The OSA-18 questionnaire has been translated and culturally adapted for use in the Turkish-speaking population [2]. Its Turkish version demonstrates excellent reliability, with a Cronbach’s alpha of 0.929. Validity is confirmed through a positive correlation between the OSA-18 score and external parameters such as the Mallampati score and tonsil and adenoid size.

Acute or chronic upper airway obstruction, resolved after tracheotomy or upper respiratory tract surgical procedures in OSA patients, can lead to severe dyspnea with pulmonary edema [3]. A 46-year-old male patient developed negative pressure pulmonary edema as a complication of tracheotomy performed due to severe upper airway obstruction following a deep neck infection, which was diagnosed early and promptly treated.

Scientometrics represents a branch of modern applied science that examines the structure and dynamics of scientific communication. Problem-oriented scientometric investigations into these aspects of science, as well as international collaboration under the conditions of globalization, competition, and institutionalization, contribute to enhancing the quality and prestige of interdisciplinary science in both powerful and smaller countries [4]. According to the authors’ concept of the unity of interdisciplinarity, institutionalization, and internationalization of science and university education [4], these essential elements of the contemporary scientific enterprise are increasingly interconnected and, therefore, should be comprehensively analyzed together.

The purpose of the present study is to analyze key scientometric features of the dynamic institutionalization and internationalization of contemporary OSA research, and to identify the leading researchers, key information sources, and prominent scientific institutions in this interdisciplinary field, thereby promoting the further development of international collaboration.

## Materials and methods

In September 2024, a retrospective, problem-oriented, title-word-based search was performed in the Science Citation Index Expanded (SCI Exp), MEDLINE, and BIOSIS Citation Index (BIOSIS) of Web of Science (Clarivate Analytics, USA), as well as in Scopus (Elsevier, the Netherlands), for the period 2019-2023.

The bibliographic descriptions of all relevant publications, whether directly or to a greater or lesser extent related to various aspects of this complex area, were retrieved and processed. The annual dynamics of the quantitative characteristics of the following essential scientometric indicators were comparatively assessed: number of abstracted publications; types of documents; languages of publication; countries of authors and their number of publications; journal titles and the number of papers abstracted in them; author names and their number of publications; thematic categories of the abstracted publications; names of scientific institutions and scientific forums, along with the number of abstracted publications; and the most productive scientific institutions. Cumulative citation parameters were also analyzed. An automated citation analysis of the most cited papers was likewise conducted.

All acquired data have been presented in the form of figures and tables using Microsoft Office 2003 software, highlighting the most prominent results.

## Results

**Figure 1:**
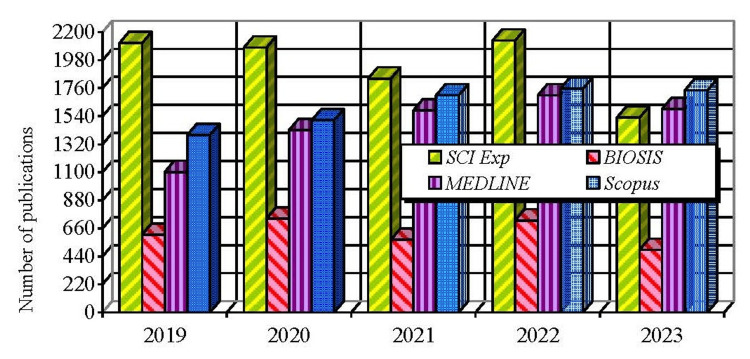
Annual trends in the number of publications abstracted in SCI Exp, Scopus, MEDLINE, and BIOSIS. SCI Exp: Science Citation Index Expanded; Scopus: Scopus Database (Elsevier, Netherlands); MEDLINE: Medical Literature Analysis and Retrieval System Online; BIOSIS: BIOSIS Citation Index (Biological Abstracts and BIOSIS Previews).

A total of 9,692 publications are abstracted in SCI Exp, 8,103 in Scopus, 7,421 in MEDLINE, and 3,130 in BIOSIS. The annual dynamics of the number of publications abstracted in these four databases are illustrated in Figure 1.

A total of 118, 165, and 74 countries have author affiliations with publications abstracted in SCI Exp, Scopus, and BIOSIS, respectively. However, there are 29, 29, and 8 countries, respectively, with only one publication by their authors.

**Figure 2:**
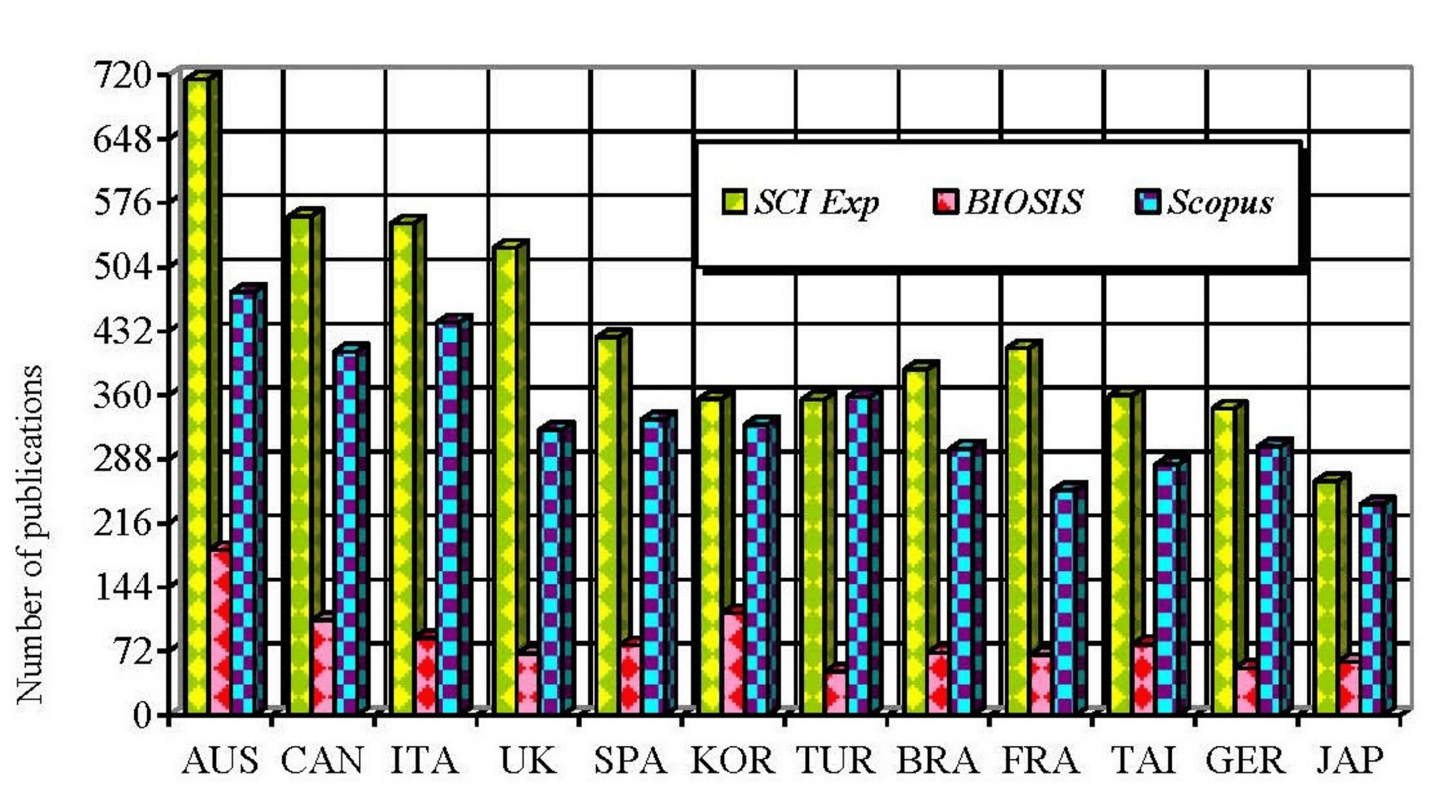
Distribution of significant countries (excluding the USA and China) by number of publications.

The significant contributions from countries (excluding the USA and China) are shown in Figure 2. Authors from the USA are represented by a total of 2,963, 1,902, and 885 publications, while those from China have 1,650, 1,558, and 503 publications abstracted in SCI Exp, Scopus, and BIOSIS, respectively.

The distributions of document types and common languages of publication are shown in Tables 1-2. There are a total of 23 publication languages: 22 in Scopus, 17 in MEDLINE, 9 in SCI Exp, and 7 in BIOSIS.

**Table 1:** Distribution of different document types across each of the mentioned databases. SCI Exp: Science Citation Index Expanded; Scopus: Scopus Database (Elsevier, Netherlands); MEDLINE: Medical Literature Analysis and Retrieval System Online; BIOSIS: BIOSIS Citation Index (Biological Abstracts and BIOSIS Previews).

Document Type	SCI Exp	BIOSIS	MEDLINE	Scopus
Article	5,155	1,867	7,020	5,955
Review	894	–	895	1,063
Letter to the Editor	413	73	329	333
Editorial	363	–	208	–
Conference Abstract	3,143	1,103	–	183
Proceedings Paper	47	–	–	184
Book Chapter	12	18	–	–
Patent	–	69	–	–
Systematic Review	–	–	379	–
Meta-analysis	–	–	369	–
Case Report	–	–	193	–
Randomized Controlled Trial	–	–	327	–
Multicentre Study	–	–	134	–
Observational Study	–	–	212	–
Comparative Study	–	109	–	–

**Table 2:** Distribution of common publication languages across each of the mentioned databases. SCI Exp: Science Citation Index Expanded; Scopus: Scopus Database (Elsevier, Netherlands); MEDLINE: Medical Literature Analysis and Retrieval System Online; BIOSIS: BIOSIS Citation Index (Biological Abstracts and BIOSIS Previews).

Language of Publication	SCI Exp	BIOSIS	MEDLINE	Scopus
English	9,964	3,099	7,385	7,479
Chinese	–	14	231	340
German	25	1	23	84
Spanish	18	4	53	65
Russian	4	10	24	88
French	12	–	30	43
Portuguese	5	–	18	21
Korean	–	–	–	15
Czech	2	–	1	11
Japanese	–	–	1	9

The journals with the highest number of papers abstracted in Web of Science, the so-called ‘core’ journals, are listed in Table 3. The journals with the most articles abstracted in Scopus include: International Journal of Pediatric Otorhinolaryngology (70), European Archives of Oto-Rhino-Laryngology (68), Annals of the American Thoracic Society (66), and Sleep Science (57 articles) [1-4].

**Table 3:** Journals with the highest number of papers abstracted in Web of Science. SCI Exp: Science Citation Index Expanded; MEDLINE: Medical Literature Analysis and Retrieval System Online; BIOSIS: BIOSIS Citation Index (Biological Abstracts and BIOSIS Previews).

No.	Journal Title	SCI Exp	BIOSIS	MEDLINE
1	Sleep	734	618	149
2	Sleep and Breathing	608	–	558
3	Journal of Sleep Medicine	718	–	346
4	Journal of Clinical Sleep Medicine	443	–	445
5	American Journal of Respiratory and Critical Care Medicine	565	91	98
6	Journal of Sleep Research	387	362	–
7	European Respiratory Journal	558	26	42
8	Chest	200	65	93
9	Scientific Reports	108	112	111
10	Journal of Clinical Medicine	148	–	151
11	Nature and Science of Sleep	126	–	127
12	European Archives of Oto-Rhino-Laryngology	85	70	83
13	PLOS ONE	75	77	78
14	The Laryngoscope	98	–	85
15	Sleep Medicine Reviews	85	–	70

The most productive authors with publications abstracted in these four databases are listed in Table 4.

**Table 4:** Most productive authors with publications abstracted in the four mentioned databases. SCI Exp: Science Citation Index Expanded; Scopus: Scopus Database (Elsevier, Netherlands); MEDLINE: Medical Literature Analysis and Retrieval System Online; BIOSIS: BIOSIS Citation Index (Biological Abstracts and BIOSIS Previews).

No.	Author’s Name	SCI Exp	BIOSIS	MEDLINE	Scopus
1	Malhotra A	125	32	76	59
2	Cistulli P	114	34	70	62
3	Pépin J	134	31	69	38
4	Gozal D	88	23	81	67
5	Eckert D	75	29	49	51
6	Redline S	67	36	46	33
7	Tufik S	63	13	53	33
8	Tamisier R	64	16	34	33
9	Keenan B	59	19	37	23
10	Peker Y	65	–	35	34
11	Penzel T	48	–	41	43
12	Sands S	74	22	33	–
13	Verbraecken J	61	–	34	30
14	Hedner J	70	29	–	22
15	Pack A	49	18	29	19

The most productive scientific institutions and their countries in SCI Exp are listed in Table 5.

**Table 5:** Most productive scientific institutions and their respective countries in SCI Exp. SCI Exp: Science Citation Index Expanded.

No.	Institution	Country	Publications
1	Flinders University	Australia	380
2	University of Pennsylvania	USA	352
3	University of California, San Diego	USA	340
4	Shanghai Jiao Tong University	China	297
5	University of Pittsburgh	USA	270
6	University of Sydney	Australia	257
7	Brigham and Women’s Hospital	USA	197
8	Stanford University	USA	172
9	University of Toronto	Canada	149
10	Harvard Medical School	USA	110
11	Chang Gung University	Taiwan	100

In Scopus, the leading institution is Beijing Anzhen Hospital, Capital Medical University (China), with 284 publications. This is followed by Chang Gung University (China) with 261, the University of Toronto (Canada) with 189, Harvard Medical School (USA) with 176, and Universiteit van Amsterdam (Netherlands) with 174 publications [1-4].

The number of events and abstracts from the most common scientific forums is shown in Table 6.

**Table 6:** Most common scientific forums based on the number of events and abstracts. WoS: Web of Science; BIOSIS: Biological Abstracts and BIOSIS Citation Index.

Forum title	WoS		BIOSIS	
	Events	Abstracts	Events	Abstracts
Annual Meeting of the Associated Professional Sleep Societies (APSS)	4	497	4	483
International Conference of the American Thoracic Society (ATS)	2	388	1	17
International Congress of the European Respiratory Society (ERS)	3	259	–	–
Congress of the European Sleep Research Society (ESRS)	1	93	1	93
Conference of the European Sleep Research Society (ESRS)	–	–	1	101
Virtual International Conference of the American Thoracic Society	1	74	–	–
Annual Scientific Meeting of the Australasian Sleep Association and the Australasian Sleep Technologists Association (ASA/ASTA)	1	62	1	62

Table 7 presents the ten most cited publications in SCI Exp/MEDLINE and Scopus [5-14].

**Table 7:** Most cited publications in SCI Exp, MEDLINE, and Scopus. SCI Exp: Science Citation Index Expanded; Scopus: Scopus Database (Elsevier, Netherlands); MEDLINE: Medical Literature Analysis and Retrieval System Online.

First Author’s Name, Ref	Brief Bibliographic Description	SCI Exp / MEDLINE	Scopus
Benjafield AV et al. [5]	The Lancet Respiratory Medicine. 2019; 7: 687-698.	1,849	2,052
Gottlieb DJ and Punjabi NM [6]	JAMA. 2020; 323: 1389-1400.	596	650
Yeghiazarians Y et al. [7]	Circulation. 2021; 144: e56-e67.	445	503
Patil SP et al. [8]	Journal of Clinical Sleep Medicine. 2019; 15: 301-334.	432	406
Veasey SC and Rosen IM [9]	The New England Journal of Medicine. 2019; 380: 1442-1449.	312	337
Patil SP et al. [10]	Journal of Clinical Sleep Medicine. 2019; 15: 335-343.	293	460
Mazzotti DR et al. [11]	American Journal of Respiratory and Critical Care Medicine. 2019; 200: 493-506.	275	294
Sánchez-de-la-Torre M et al. [12]	The Lancet Respiratory Medicine. 2020; 8: 359-367.	262	276
Tietjens JR et al. [13]	Journal of the American Heart Association. 2019; 8: e010440.	221	235
Fietze I et al. [14]	Journal of Sleep Research. 2019; 28: e12770.	199	224

Table 8 displays the cumulative citation parameters in SCI Exp and BIOSIS. The values of the Hirsch index are relatively high.

**Table 8:** Cumulative citation metrics in SCI Exp and BIOSIS. BIOSIS: BIOSIS Citation Index (Biological Abstracts and BIOSIS Previews); SCI Exp: Science Citation Index Expanded.

Citation Parameter	SCI Exp	BIOSIS
Total number of abstracted publications	9,692	3,130
Total number of received citations	58,307	7,775
Total number of received citations (excluding self-citations)	37,095	5,334
Percentage of citations without self-citations (%)	63.62	68.6
Total number of citing articles	21,284	4,604
Total number of citing articles (excluding self-citing articles)	16,667	3,620
Percentage of citing articles without self-citation (%)	78.31	78.63
Average number of received citations per article	6	2.48
Number of articles cited at least once	5,910	1,399
Percentage of articles cited at least once (%)	60.98	44.7
Hirsch index (h-index)	69	32

Clinical neurology, respiratory system, and neurosciences are among the most common thematic categories in SCI Exp, while complications, polysomnography, diagnosis, therapy, and epidemiology of OSA are predominant in the Medical Subject Headings in MEDLINE.

## Discussion

Our results are similar to recently published scientometric investigations on this hot topic.

A retrospective, problem-oriented scientometric investigation of the use of geographical information systems in health planning across six databases between 1990 and 2010 reveals that MEDLINE (via PubMed) presents with 484 papers published in 243 journals [15]. Publications by authors from 44 countries are abstracted in Web of Science. English dominates, with 14 other languages represented in significant numbers.

The results of a retrospective, problem-oriented scientometric study on pediatric sleep apnea in Web of Science, MEDLINE, and Scopus show a steady increase in global publication output [16]. Between 1985 and 2010, more than 8,100 authors from 64 countries published 3,213 papers in 626 journals and 256 conference proceedings abstracted in Web of Science. Between 1973 and 2010, 152 authors published 687 papers in 144 journals, in 19 languages, abstracted in Scopus.

A bibliometric analysis of 4,022 publications on childhood OSA abstracted in Web of Science and PubMed between 2013 and 2022 shows that the USA presents with the highest number of publications [17]. The University of Cincinnati and the University of Pennsylvania are the most productive organizations. The International Journal of Pediatric Otorhinolaryngology is the most prolific journal.

One bibliometric analysis of 1,995 articles devoted to OSA and cognition, retrieved from Web of Science between 2003 and 2022, demonstrates a notable increase in publication numbers and significant contributions from authors and institutions in the USA, particularly Harvard University [18]. Another analysis of 79 articles dealing with OSA and anesthesia identifies the following hot topics: airway safety, postoperative pain control, and noninvasive mechanical ventilation treatments such as continuous positive airway pressure [19].

Co-citation and cluster analyses, along with keyword co-occurrence of relevant publications extracted over the last 20 years from the Web of Science Core Collection, identify the most prominent terms related to sleep quality and associated genes: OSA, genome-wide association studies, circadian rhythms, DNA methylation, and depression [20].

A bibliometric analysis of 1,894 publications devoted to postoperative pain and sleep disorders retrieved from the Web of Science database between 2013 and 2023 confirms that OSA is a hot topic in this field [21]. A global collaborative network is centered around the USA, China, and Europe.

Bibliometric analyses and science mapping of 7,263 articles and reviews addressing the association between arterial hypertension and OSA, searched in the Web of Science Core Collection up to July 3, 2021, identify rapid research development in this interdisciplinary field [22]. The USA and Harvard Medical School dominate, and Sleep and Breathing is the most productive journal.

Between 2009 and 2018, a total of 12,666 articles on OSA were abstracted in SCI Exp of Web of Science [23]. Their number increased from 895 in 2009 to 1,592 in 2018. The international collaboration rate was 18.2%. The country collaboration network identifies the USA as the hegemonic node.

An analysis of 24,291 compliant publications on OSA retrieved from Web of Science between 2011 and 2020 demonstrates a consistent annual increase in volume [24]. The USA dominates global research in this field. Gozal D has made significant contributions, and the University of Pennsylvania is the most prolific scientific institution.

Bibliometric and visualization analyses of 2,584 publications on OSA treatment in adults, retrieved from the Web of Science Core Collection between 1999 and 2022, indicate the dominance of the USA and the University of Sydney [25].

A bibliometric analysis of the relationship between OSA and cardiovascular diseases reveals a total of 7,047 publications abstracted in the Web of Science Core Collection between 2010 and 2021 [26]. The USA is the largest contributor and a key player in the international cooperation network.

Among a total of 42,878 manuscripts on OSA abstracted in Web of Science, 100 influential manuscripts were published between 2005 and 2017, mainly in The American Journal of Respiratory and Critical Care Medicine, Sleep, and Chest [27]. In the same database, 14 OSA-related papers were bibliometrically identified between 1992 and 2018 among the top 100 most-cited randomized controlled trial reports in the field of orthodontics [28]. Additionally, the top 100 most cited articles in sleep medicine, published in 49 journals by institutions from nine countries, include 22 focused directly on OSA, with Sleep being the most frequently cited journal [29].

The University of Helsinki, the University of Heidelberg, and the German Cancer Research Center were identified in the Web of Science Core Collection, MEDLINE, and BIOSIS Citation Index of Web of Knowledge, as well as in Scopus, between 1987 and 2016, as the most influential scientific institutions in the field of colorectal tumour markers [30].

## Conclusions

Authors’ analysis identifies the recent contributions of individual countries, researchers, and their institutions in OSA. The outlined dynamic science stratification on this topic, concerning publication output, citation practices, and international collaboration, deserves special attention from science policymakers and research managers in smaller countries. This rich bibliographic data collection can be used by clinicians and scientists striving for greater international visibility and prestige for their national medical science.

The syndrome of OSA and snoring is one of the serious health issues of the 21st century. It severely affects the quality of life of those impacted. Therefore, adequate and timely measures, both medical and legislative, must be taken to address its consequences.
